# Insights on the Horizontal Gene Transfer of Carbapenemase Determinants in the Opportunistic Pathogen *Acinetobacter baumannii*

**DOI:** 10.3390/microorganisms4030029

**Published:** 2016-08-23

**Authors:** Gabriela Jorge Da Silva, Sara Domingues

**Affiliations:** 1 Faculty of Pharmacy, University of Coimbra, Coimbra 3000-458, Portugal; saradomingues@ff.uc.pt; 2 Centre for Neuroscience and Cell Biology, University of Coimbra, Coimbra 3000-458, Portugal

**Keywords:** *Acinetobacter*, oxacillinases, metallo-β-lactamases, conjugation, transduction, natural transformation, outer-membrane vesicles-mediated transfer, epidemiology, antimicrobial resistance

## Abstract

Horizontal gene transfer (HGT) is a driving force to the evolution of bacteria. The fast emergence of antimicrobial resistance reflects the ability of genetic adaptation of pathogens. *Acinetobacter baumannii* has emerged in the last few decades as an important opportunistic nosocomial pathogen, in part due to its high capacity of acquiring resistance to diverse antibiotic families, including to the so-called last line drugs such as carbapenems. The rampant selective pressure and genetic exchange of resistance genes hinder the effective treatment of resistant infections. *A. baumannii* uses all the resistance mechanisms to survive against carbapenems but production of carbapenemases are the major mechanism, which may act in synergy with others. *A. baumannii* appears to use all the mechanisms of gene dissemination. Beyond conjugation, the mostly reported recent studies point to natural transformation, transduction and outer membrane vesicles-mediated transfer as mechanisms that may play a role in carbapenemase determinants spread. Understanding the genetic mobilization of carbapenemase genes is paramount in preventing their dissemination. Here we review the carbapenemases found in *A. baumannii* and present an overview of the current knowledge of contributions of the various HGT mechanisms to the molecular epidemiology of carbapenem resistance in this relevant opportunistic pathogen.

## 1. Introduction

Antimicrobial resistance has become a public health problem at a global scale; it limits therapeutic options and thereby increases morbidity, mortality and treatment costs. The emergence of resistance in pathogens and its dynamics in the last decades is the result of an evolutionary process, in part fueled by anthropogenic activities. Resistance can arise by mutation, transmitted vertically through populations by cell division, or through the acquisition of resistance gene(s) from other bacteria belonging to the same generation in a process called horizontal gene transfer (HGT). HGT is recognized as a major evolutionary force that is constantly reshaping genomes. Three principal mechanisms of intercellular transfer are described: natural transformation (transfer of naked DNA to a recipient cell), transduction (phage assisted transfer) and conjugation (direct cell to cell transfer through conjugative plasmids); the latter most often associated with the dissemination of resistance [1]. Additionally, resistance genes can be mobilized by transposable elements within the chromosome and to plasmids (intracellular movement) and vice-versa.

*Acinetobacter baumannii* emerged as an important opportunistic pathogen with a high ability to acquire antimicrobial resistance and nowadays, many strains are only susceptible to carbapenems and colistin. However, the finding of carbapenem-resistant strains have been increasing worldwide [2]. As other bacteria, *A. baumannii* contains plasmids and conjugation can undoubtedly explain the dissemination of certain type of carbepenem-resistant genes, such as some OXA-carbapenemases [3,4]. Nevertheless, finding identical integrons with the same resistance genes cassettes arrays in genetically unrelated *A. baumannii* isolates lacking plasmid srules out the paradigm of conjugation as the major driving force in acquisition of exogenous DNA in this species [5]. To gain insights into the intraspecies diversity and the epidemiology of resistance genes it is important to understand the molecular mechanisms underlying the flux of resistance genes among bacteria. Due to the expanded ability of genetic adaptation, the clinical importance and emergent carbapenem resistance of this species, we review the most common carbapenemases produced by *A. baumannii* and the lateral transfer mechanisms of carbapenemase genes involved in their dissemination, in a species that uses all the classic mechanisms referred above and also the more recently identified outer membrane vesicles (OMVs)-mediated transfer.

## 2. Clinical Importance of *Acinetobacter baumannii*

Members of the genus *Acinetobacter* are considered ubiquitous microorganisms. Its taxonomy has been evolving with the development of molecular and sequencing methods. Species other than *A. baumannii* are found in environmental water and are colonizers of the human skin in some individuals, but not *A. baumannii*, whose habitat remains undefined. Nevertheless, hospital patients may become colonized by this species. It is by far the most clinically important species of *Acinetobacter* as an opportunistic pathogen and it is mostly implicated in infections in critically ill patients hospitalized at Intensive Care Units (ICU). The most frequent hospital-acquired infections by *A. baumannii* are ventilator-associated pneumonia and bloodstream infections, with a considerable morbidity and mortality associated. Other infections include skin and soft tissues infections (such as in burnt patients), wound infections, urinary tract infections and more rarely, secondary meningitis [6].

Therefore, *A. baumannii* has emerged as one of the more problematic opportunistic pathogens in the clinical settings, especially in the last 20 years, now being included in the group of microorganisms which show a high antibiotic resistance index, designated by the acronym ESKAPE (*Enterococcus faecium*, *Staphylococcus aureus*, *Klebsiella pneumoniae*, *A. baumannii*, *Pseudomonas aeruginosa*, *Enterobacter* spp.). Its clinical significance is mostly associated with the ability to quickly acquire antimicrobial resistance to different classes of antibiotics [7]. Additionally, it is one of the Gram-negative bacilli that shows an incredible ability to survive on dry surfaces for prolonged periods, which potentiates its dissemination in the nosocomial environment [8,9].

## 3. Evolution of Resistance towards Large Spectrum Antibiotics

The absence of large surveillance studies between the 70s and 90s makes the objective analysis of the evolution of resistance of this microorganism and comparison between regions difficult. Moreover, the problem of resistance might be associated with the dissemination of specific clonal lineages. For instance, in Portugal, the first nosocomial isolates of *Acinetobacter* spp. collected in the 1970s–1980s were only resistant to aminopenicillins, first and some second-generation cephalosporins [10]. Also, the identification was not precise and some isolates could not be *A. baumannii*, the prominent species in hospital environments nowadays. After 1999, the scenario changed dramatically with the majority of the isolates showing only susceptibility to tobramycin, amikacin and colistin, which was associated with the European Clone II disseminated all over the country [11,12,13]. Other European countries reported a similar increase in the isolation of multidrug resistant *A. baumannii* [6,14,15,16]. It is unquestionable that *A. baumannii*, or at least some lineages [17], have a remarkable ability to acquire resistance determinants and, though its image of a low-virulence pathogen, it cannot be underestimated [18,19]. Often, it is resistant to the majority of classes of antibiotics, including carbapenems, and sometimes colistin, considered the last therapeutic resources for infections caused by multidrug-resistant Gram-negative bacteria [20,21].

## 4. Carbapenem Resistance in *Acinetobacter baumannii*

Carbapenems (imipenem, meropenem, biapenem, ertapenem, and doripenem) are the β-lactam antibiotics with the broadest spectrum of activity, showing great activity against Gram-negative and Gram-positive bacteria and are considered “last line agents”. Also, they are relatively stable to the majority of β-lactamases, the more frequent reported mechanism of resistance to β-lactam antibiotics. Nevertheless, the resistance to carbapenems has been dramatically increasing over recent years among the Gram-negatives [22,23].

Diverse other mechanisms may be responsible for carbapenem resistance in *A. baumannii*. The loss or alterations of specific outer-membrane proteins have been reported to play a role in carbapenem resistance (CarO) [24]. Occasional reports associated the resistance to carbapenems to modification of penicillin binding proteins (PBPs) [25]. The AdeABC efflux pump, belonging to the resistance nodulation division family of efflux systems (RND) and associated with the efflux of antibiotics with diverse structures [26], was shown to be present in *A. baumannii* [27,28] and eventually to play some role in carbapenem resistance. However, the latter resistance mechanism appeared to give clinical resistance to carbapenems only when associated with others, namely the production of carbapenem-hydrolysing oxacillinases [29,30].

## 5. Production of Carbapenemases

### 5.1. Intrinsic β-Lactamases

*A. baumannii* produces intrinsic β-lactamases such as an AmpC-type cephalosporinase expressed at low levels. However, when the insertion sequence IS*Aba1* is located upstream the *bla*_AmpC_ gene, its expression is enhanced and provides resistance to 3rd generation cephalosporins, but not to carbapenems. 

Additionally, it seems that the majority of *A. baumannii* strains produce oxacillinases represented by OXA-51/69 variants [31,32]. These enzymes show weak hydrolytic activity towards carbapenems. However, isolates bearing the IS*Aba1*-*bla*_OXA-51_-like gene, show higher rates of resistance to imipenem and meropenem [33,34]. The *bla*_OXA-51_-type are usually located in the chromosome. It has been suggested its use as a marker for identification of the species [35] but it has also been shown that other species may eventually carry this oxacillinase in plasmids [36,37]. Detection of target site duplications surrounding transposon Tn*6080*, which carry the *bla*_OXA-51_-like gene in *A. baumannii* plasmids, suggest that this genetic element has moved by transposition from the chromosome of this species [34]. Transfer of plasmid-carrying *bla*_OXA-51_-like by HGT, probably conjugation, to non-*baumannii* species has probably occurred. Conjugation of plasmids carrying the *bla*_OXA-51_-like gene from *Acinetobacter nosocomialis* to *A. baumannii* was observed [37]. 

### 5.2. Acquired β-Lactamases

The most striking activity against to carbapenems in *A. baumannii* has been described by the acquisition of diverse carbapenemase encoding-genes. Carbapenemases are a group of enzymes that are able to hydrolyse carbapenems even at low level. The emergence of infections caused by extended spectrum β-lactamases (ESBLs) Gram-negative producers in the early 1990s, concomitantly resistant to other classes of antibiotics such as quinolones and aminoglycosides, lead to the more frequent use of carbapenems in complicated infections, which may be associated with the emergence and spread of carbapenemases through all continents [38,39,40].

Carbapenemases belong to three classes of the Ambler classification [41]: the class B, comprising the metallo-β-lactamases (MBLs) that require a bivalent metal ion, usually Zn^2+^, for activity; and the class D, the typical class of oxacillinases, and to class A. The former are potent carbapenemases with the ability to hydrolyse all β-lactams, except aztreonam. To date, three families of MBLs have been identified in *A. baumannii*: the IMP-, the VIM- and NDM-types. Although the *bla*_SIM-1_ gene was described for the first time as part of the class 1 integron present in seven clinical isolates of *A. baumannii* in 2005 [42], the isolates were later identified as *A. pittii* [43]. So far, there are no reports on the presence of this gene in *A. baumannii.* Oxacillinases are a heterogeneous group of β-lactamases with higher efficiency in the hydrolysis of oxacillin than benzylpenicillin [44]. Only a sub-group of oxacillinases have the ability of inactivating carbapenems, usually not as efficiently as MBLs and are named carbapenem-hydrolysing class D β-lactamases (CHDLs). The acquired sub-group of CHDLs in *A. baumannii* can be clustered in: OXA-23-like, OXA-40/24-like; OXA-58-like [3]; OXA-143-like [45]; and OXA-235-like [46] (Table 1). To our knowledge, only one study reports the finding of the *bla*_OXA-48_ gene in *A. baumannii.* However, identification of the location and genetic environment of the gene was not the focus of this report [47].

Both MBLs and CHDLs are resistant to the β-lactam inhibitors clavulanic acid and tazobactam [44].

## 6. Mechanisms of Acquisition of Carbapenemase Determinants in *Acinetobacter baumannii*

Despite the numerous reports on the mechanisms of the acquisition of exogenous DNA by bacteria, our knowledge is far from complete in explaining the rapid genetic evolution of bacteria. Some species seem more prone to acquiring exogenous DNA than others. *A. baumannii* has undoubtedly an enormous genetic plasticity in order to survive. 

Members of the *Acinetobacter* genus can exploit four different HGT mechanisms to exchange genetic material. Conjugation is reported to have the greatest impact in the dissemination of antimicrobial resistance [48]. Transduction and transformation are apparently less important, yet recent studies suggest that their role may be more expressive than initially thought.

All of the HGT mechanisms have been specifically observed in *A. baumannii.* Many of the *bla*_OXA_ genes and some MBLs determinants, such as *bla*_NDM_ genes, are found to be plasmidic, suggesting that conjugation has an important role in the dissemination of these carbapenemases determinants. Nonetheless, a few studies failed to demonstrate conjugation of plasmids in this species [49,50,51,52] and quite often their dissemination is inferred from their plasmid location, especially in epidemiological studies where conjugation assays are not demonstrated. Moreover, the isolation and study of *Acinetobacter* plasmids has been shown to be a difficult task, which may be a limiting factor in experimental assays [52,53]. A few studies have experimentally shown transduction in *A. baumannii* [54,55]. Natural transformation is known to occur in the *Acinetobacter* genus [56]. However, this mechanism has not been demonstrated to occur in clinical *A. baumannii* isolates until recently. Indeed, two studies and our own current research identified naturally competent strains of *A. baumannii*; this species can under certain conditions take up both chromosomal and plasmid DNA [57,58], leading to the hypothesis that natural transformation may have some influence in the dynamics of resistance genes in *A. baumannii*. Another mechanism was also demonstrated in *A. baumannii*, where plasmid DNA transfer can be mediated by OMVs [59], a mechanism not frequently reported in explaining the dissemination of resistance genes and most probably underestimated.

The next chapters will review the mechanisms of the horizontal transfer of carbapenemase genes in *A. baumannii*. Unlike the majority of oxacillinases in other genera, none of the *A. baumannii* *bla*_OXA_ genes associated with carbapenem resistance have been found in integrons [2,60]. On the other hand, some MBLs are embedded as gene cassettes in integrons, which are not self-mobile but are often inserted into mobile elements [2,61].

### 6.1. Mechanisms Involved in the Movement and Dissemination of CHDLs

#### 6.1.1. OXA-23 (and OXA-23-Like)

The *bla*_OXA-23_ (previously known as *bla*_ARI-1_) gene, identified for the first time in a clinical *A. baumannii* isolate collected in 1985 [62,63], can be inserted both in the chromosome and in plasmids and be surrounded by different genetic contexts, such as genomic islands (AbaR4, AbaR25 and AbaR26), transposons (Tn*2006*, Tn*2007*, Tn*2008*, Tn*2009* and Tn*6206*) and insertion sequences (ISs) [64,65,66,67]. 

The *bla*_OXA-23_ gene, which is nowadays widely distributed in *A. baumannii,* was originally naturally present in *Acinetobacter radioresistens* strains, though not expressed and a sequence of different transposition and horizontal transfer events have probably resulted in the transfer of this gene to *A. baumannii* [66,121]. This gene has been identified in other *Acinetobacter* species, namely *Acinetobacter baylyi* [122], *Acinetobacter johnsonii* [123], *Acinetobacter gandensis* [124], *Acinetobacter* genomic species 14TU/13BJ [125], *Acinetobacter* genomic species 15TU [53], *A. nosocomialis* [125] and *Acinetobacter pittii* [123]. The same genetic context of the *bla*_OXA-23_ gene in *A. baumannii* is found in some of these species, suggesting occurrence of HGT [53,67,122,125,126]. There are also three reports on the detection of the *bla*_OXA-23_ gene in two species of the *Enterobacteriaceae* family, namely *Escherichia coli* [127] and *Proteus mirabilis* [128,129]. While the *bla*_OXA-23_ gene in *P. mirabilis* is chromosomally-located [128,129], the gene found in *E. coli* is flanked by two IS*1* elements and it is located on a non-self-conjugative plasmid, which failed conjugation to *E. coli* and *A. baumannii* recipient strains [127]. 

Expression of this gene in *A. baumannii* is associated with the presence of IS*Aba* insertion sequences, especially IS*Aba1*, preceding it [33,66,68,69]; the association of IS*Aba1* with *bla*_OXA-23_ has also been implicated in the movement of this gene by transposition, as experimentally shown [70] or as reflected by the identification of target site duplications surrounding the composite-transposons [66,67,69]. 

Horizontal transfer of this gene due to plasmid conjugation between *A. baumannii* strains has been demonstrated [64,71,72]; conjugation of a plasmid-carrying *bla*_OXA-23_ from *A. baumannii* to *A. baylyi* [130] has also been shown. Intracellular movement of transposons-carrying the *bla*_OXA-23_ gene by transposition can be suggested due to the identification of target site duplications surrounding the transposons, as determined by nucleotide sequencing of the genetic context of the gene in wildtype *A. baumannii* strains [64,71,73]; the same is valid for genomic islands [72]. 

Evidence of HGT has also been inferred from the different chromosomal insertion sites of the *bla*_OXA-23_ gene in isolates belonging to the same clonal complex [130]. Other evidence demonstrating the horizontal movement of the *bla*_OXA-23_ gene associated with the IS*Aba1* mobile element has come from the fact that the same genetic context is detected in several genetically unrelated isolates [68].

#### 6.1.2. OXA-40/24 (and OXA-40-Like)

The *bla*_OXA-40_ (firstly identified as *bla*_OXA-24_) gene was identified for the first time in a clinical *A. baumannii* strain isolated in 1997 [74,131]. The *bla*_OXA-40_ and *bla*_OXA-40_-like genes reported in *A. baumannii* are located both in the chromosome [74,75,76] and in plasmids [45,76,77]. However, experimental demonstration of conjugation of *A. baumannii* plasmid-encoding *bla*_OXA-40_-like genes into *A. baumannii* and *E. coli* have failed [76,78]. Nonetheless, the presence of this gene in plasmids belonging to the replicon group GR6, which are self-transmissible and in plasmids containing *mob* genes, which can be mobilized by self-transmissible plasmids, indicates a potential for horizontal dissemination by conjugation [52,78]. The lower GC content of the *bla*_OXA-40_ gene, as compared with the remaining genome of *A. baumannii,* suggests a different species as the source of this gene [3].

The *bla*_OXA-40_ gene found in the *A. baumannii* chromosome or in different plasmids is not embedded into the typical structures involved in DNA mobilization such as ISs but is instead flanked by conserved inverted repeats homologous to XerC/XerD binding sites [77,79,80]. These sites are the targets of the XerC and XerD recombinases, which are involved in site-specific recombination mechanisms. It is suggested that the *bla*_OXA-40_ gene can be mobilized by this mechanism within the *A. baumannii* isolates and then further spread by HGT. This hypothesis is supported by the fact that the same structure is located in different plasmids and in the chromosome [77,78] and that the same binding sites flank different DNA modules in other locations [79]. Identification of genes involved in the toxin/antitoxin system in plasmids harboring the *bla*_OXA-40_ gene might explain the wide dissemination and stability of the plasmids containing this gene [132].

This gene has been occasionally reported in other *Acinetobacter* species such as *A. baylyi* [133], *Acinetobacter calcoaceticus* [77], *Acinetobacter haemolyticus* [134] and *A. pittii* [125]. The same plasmid containing the *bla*_OXA-40_ gene flanked by the XerC/XerD binding sites has been found in *A. baumannii, A. calcoaceticus* [77], *A. baumannii* and *A. haemolyticus* [78], which suggests transfer of this genetic element from *A. baumannii* to the other species, by a HGT mechanism that has not yet been determined. The *A. baumannii bla*_OXA-40_ gene has also been detected in *P. aeruginosa*, in a plasmid similar to one found in *A. baumannii* [135].

OMVs of Gram-negative bacteria serve a wide number of biological functions and have been extensively studied in the delivery of proteins and toxins in order to target cells during infection, and therefore, are mostly associated with virulence factors [136]. Nevertheless, Rumbo and colleagues [59] have experimentally shown the release of OMVs containing the plasmid-encoded *bla*_OXA-24_ from carbapenem-resistant *A. baumannii* clinical strains that were able to transform a carbapenem-susceptible *A. baumannii* strain. The transformants maintained the capacity to release OMVs containing the plasmid-encoded *bla*_OXA-24_. The OMVs-mediated transfer remains largely unexplored in the spread of antimicrobial resistance and this study has been the first experimental evidence that clinical isolates of *A. baumannii* may release OMVs carrying carbapenemase determinants as a mechanism of HGT. 

#### 6.1.3. OXA-58 (and OXA-58-Like)

The *bla*_OXA-58_ gene was identified for the first time in 2003 in a clinical *A. baumannii* isolate [120]. This gene is usually plasmid-encoded and it is found in different plasmids [67,81,82]. Despite the plasmid location, several attempts have failed to demonstrate conjugation of this genetic mobile element between *A. baumannii* or from *Acinetobacter* spp. to *A. baumannii* strains [50,51,120]. Therefore, the involvement of natural transformation, transduction and/or OMVs-mediated transfer could eventually explain the wide spread of the plasmid-encoded *bla*_OXA-58_ gene. 

The existence of similar plasmid-carrying *bla*_OXA-58_ in unrelated strains and with different origins (clinical vs. hospital environment) is seen has evidence of the HGT of this element [51]. Although sporadically, this gene has also been detected in other *Acinetobacter* species, including *Acinetobacter bereziniae* [137], *Acinetobacter guillouiae* [138], *A. johnsonii* [139], *Acinetobacter junii* [81], *Acinetobacter lwoffii* [137], *A. nosocomialis* [50], *Acinetobacter phenon* 6/ct13TU [140], *A. pittii* [50,141], *A. radioresistens* [142] and *Acinetobacter seifertii* [143]. One recent study has observed a possible mobilization of a plasmid-encoding *bla*_OXA-58_ during conjugation of a self-transmissible plasmid from *A. pittii* to *A. baumannii* [144]. It has been suggested that in *A. baumannii* plasmid-encoding, *bla*_OXA-58_ can be mobilized by the self-conjugative plasmids belonging to the replicon group GR6 [52,83].

In *A. baumannii* the *bla*_OXA-58_ gene is surrounded by IS*Aba3* [3] however the upstream IS*Aba3*-like element is often disrupted by different ISs such as IS*Aba1*, IS*Aba2* [84], IS*Our1*, IS*1008*, IS*15* [50] and IS*Aba825* [85], which enhance the expression of the *bla*_OXA-58_ gene. Despite the fact that the *bla*_OXA-58_ gene is surrounded by these mobile elements and that similar *bla*_OXA-58_ genetic contexts are found in different *Acinetobacter* species and strains [50,84,141], there is little evidence that this structure has moved by transposition [3]. Rather, it is proposed that the acquisition of the *bla*_OXA-58_ gene and respective genetic context resulted from homologous recombination events, evidenced by the fact that different genetic structures containing the gene are flanked by two repeated sequences called Re27 [84]. The same recombination sites and the *bla*_OXA-58_ gene and respective gene surrounding has been identified in *A. nosocomialis* and *A. pittii* [50]. There is only one study that suggests that the *bla*_OXA-58_ gene flanked by an IS*Aba3* and an intact IS*Aba3*-like elements as transposed into a plasmid in *A. pittii* [144]. The duplication of an IS*Aba2*/IS*Aba3*-*bla*_OXA-58_-IS*Aba3* unit in clinical *A. baumannii* isolates has been linked to the action of two IS*26* elements surrounding the previous unit; multiple copy numbers of the *bla*_OXA-58_ gene was correlated with increased Minimum Inhibitory Concentrations of carbapenems [86].

It has been suggested that the *bla*_OXA-58_ gene widely disseminated among *A. baumannii* strains has its source in a different species, reflected by the different GC content of the gene, when compared with its core genome [3]. However, the source has not been identified so far.

Recently, the mechanism of dissemination of OXA-58 CHDL was elucidated. Not being exactly a mechanism of gene transfer, the formation of OMVs by *A. baumannii* also contributed to the release of extracellular OXA-58 CHDL. OXA-58 is selectively released via OMVs after using the Sec-dependent periplasmic translocation transport system and its release was increased upon a carbapenem challenge [145]. These OMVs have a sheltering effect on carbapenem-susceptible bacteria allowing the unexpected survival of the cells in, for example, polimicrobial infections [145,146]. Yet not sharing genetic material, the overexpression of OXA-58 by *A. baumannii* protects other bacteria. However, as in the case of the transfer of *bla*_OXA-40_ OMVs, the genetic exchange cannot be discarded.

#### 6.1.4. OXA-143 (and OXA-143-Like)

The OXA-143 sub-group of CHDLs was described for the first time in *A. baumannii* in 2009; the *bla*_OXA-143_ gene was plasmid located and flanked by two copies of the same replicase gene, suggesting acquisition by homologous recombination [45].

One *bla*_OXA-143_-like gene, *bla*_OXA-253_, was also detected as part of a plasmid that resembles one plasmid carrying the *bla*_OXA-40/24_ gene; acquisition of the *bla*_OXA-253_ gene is suggested to have occurred by site-specific recombination in the XerC/XerD site [87].

All isolates reported to carry a *bla*_OXA-143_-like gene so far were isolated in Brazil [45,87,147,148,149].

None of the published studies have performed HGT assays that could be involved in the dissemination of these CHDLs. However, horizontal transfer might be inferred from the presence of the same gene in diverse genotypes [147].

#### 6.1.5. OXA-235 (and OXA-235-Like)

Recently, a new subclass of CHDL was identified, the OXA-235. The *bla*_OXA-235_ gene has been found both in the chromosome and in plasmids. These genes have been found flanked by two IS*Aba1*, suggesting that they can potentially move by transposition [46].

### 6.2. Mechanisms Involved in the Movement and Dissemination of MBL Genes

Some MBLs are inserted in integrons that may be integrated in mobile genetic elements such as transposons; some are chromosomal, others are located in plasmids, which may more easily explain their dissemination. Integrons are genetic elements that can capture gene cassettes by site-specific recombination. Any gene cassette embedded in an integron that enters a cell by HGT mechanisms can potentially be recruited by an already existing integron [150] but this has not been experimentally demonstrated with MBL-encoding genes. The MBL-encoding genes present in *A. baumannii* can be found in a variety of different cassette arrays (http://integrall.bio.ua.pt/).

#### 6.2.1. IMP-Type

From the 52*bla*_IMP_ gene variants that have been described [151], at least 12 have been detected in *A. baumannii* [2,88] (GenBank accession number KT935306). 

The *A. baumannii* class 1 integrons carrying *bla*_IMP_ genes can be chromosomally- [89,90,91] or plasmid-located [88,92,93]. Class 1 integrons carrying *bla*_IMP_ genes are also often found in Enterobacteriaceae, usually in transferable plasmids [61,152], which does not seem to be the case in *A. baumannii* [92,152]. Conjugation of plasmids-carrying the *bla*_IMP-5_ gene from *A. bereziniae* to *A. baumannii* has also been unsuccessful [153]. Conjugation of a *bla*_IMP_ gene between *A. baumannii* strains has been reported by Takahashi and colleagues [154]. However, the authors did not detect plasmids in their donor isolates, often difficult to isolate in this species, but it opens up the possibility of the transfer of the *bla*_IMP_ gene by another HGT mechanism. Nonetheless, *bla*_IMP_ genes are not always embedded in integrons and conjugation of a plasmid-carrying the *bla*_IMP-1_ gene not associated with class 1 integron from *A. baumannii* to *E. coli* was reported [94]. 

The presence of the same class 1 integron in different bacterial species and genus is seen as an evidence of HGT [91,150,152].

Evidence of the intracellular movement by transposition of a class 1 integron-carrying the *bla*_IMP-5_ gene flanked by miniature inverted repeat transposable elements (MITEs) in *A. baumannii* can be inferred from the determination of target site duplications on both sides of the MITEs [95]; identical MITEs were found flanking the integron-carrying *bla*_IMP-1_ gene in *A. baumannii* isolates [96]. 

Intercellular transfer of chromosomally-located genes cannot be explained by conjugation. So far, there are no reports on the dissemination of carbapenem-resistance genes by natural transformation between *A. baumannii* strains, which could be explained by the fact that only very recently has this species been shown to be competent in specific conditions [57,58]. Nonetheless, the hypothesis that *A. baumannii* could be a source for carbapenem-resistance genes dissemination due to natural transformation events cannot be neglected. It was experimentally demonstrated that the chromosomal class 1 integron-carrying *bla*_IMP-5_ of the clinical *A. baumannii* 65FFC strain [89] was transferred by natural transformation to the integron-carrying *A. baylyi* SD2 (derivative of strain BD413) and incorporated into the recipient’s chromosome by homologous recombination of the conserved regions of this class of integron [97]. From this study we might infer that carbapenem-resistance genes incorporated into class 1 integrons can be potentially acquired by all natural competent species that do not restrict the DNA uptake to the species level. 

#### 6.2.2. VIM-Type

Forty-eight *bla*_VIM_ gene variants have been described so far [151]; among these, only six have been reported in *A. baumannii* [2]. The *bla*_VIM_ genes embedded into class 1 integrons have been identified in a limited variety of gene cassette arrays in *A. baumannii* and are not frequently reported in this species; reports on these genes are more abundant and diverse in *P. aeruginosa* [155,156]. The *bla*_VIM-2_ gene has also been identified in a class 2 integron in *A. baumannii* (GenBank accession number LC107606). 

Most of the studies do not report on the genetic environment of the class 1 integrons carrying the *bla*_VIM_ genes [98,99,100], which hinders the unravelling of the horizontal mechanisms involved in their dissemination; these have been described in the chromosome of *A. baumannii* but without further analysis of the genetic context [101,102]. Up to now, there have been no studies showing the horizontal transfer of VIM determinants in *A. baumannii.* Horizontal transfer of VIM-encoding genes can be inferred from the presence of the same integron in genetically non-related *A. baumannii* isolates [98,157], in different species and genus [158]. Conjugation of *bla*_VIM-1_ genes from *A. baumannii* to *E. coli* has failed. However, it is not clarified if this failure is due to chromosomal location of the gene or due to inability of the plasmid to conjugate [98]. 

#### 6.2.3. NDM-Type

Since the identification of the New Delhi metallo-β-lactamase (NDM) in 2008 in India, this MBL has been reported worldwide. The *bla*_NDM_ genes have been recognized in a variety of species and genus and its location in diverse replicon type plasmids, such as IncA/C2 and IncFIIY plasmids, is the most likely mechanism in explaining this expanded dissemination [103]. Some examples are further mentioned.

Until now, 16 variants of the *bla*_NDM_ gene have been identified [151]. Three of these variants, namely *bla*_NDM-1_, *bla*_NDM-2_ and *bla*_NDM-3_ were identified in *A. baumannii* [159,160] (GenBank accession number KU220611). 

Interestingly, a clinical case in China reports the finding of *bla*_NDM-1_ in *E. coli*, *Citrobacter freundii* and *A. baumannii* collected from the same patient but while *bla*_NDM-1_ was inserted in an identical plasmid in the Enterobacteria, in *A. baumannii* it was found to be located in a very large plasmid (>400-Kb) [104]. The genetic environment was not analysed. The *bla*_NDM-1_ determinant was also located in a plasmid of 100-Kb in an isolate from Brazil [105]. In Switzerland, the *bla*_NDM-1_ gene has been found in Enterobacteria located on conjugative IncA/C- or IncF-type plasmids.In all strains, part of IS*Aba125,* previously identified in *bla*_NDM-1_-negative *A. baumannii,* was found upstream of the *bla*_NDM-1_ gene, suggesting that the original dissemination occurred from *A. baumannii* [106]. This carbapenemase can also be found in animals. An isolate of *A. baumannii* positive for *bla*_NDM-1_ was collected from a lung sample of a pig with pneumonia and sepsis. The *bla*_NDM-1_ gene was located in a ~47-Kb plasmid. This plasmid could be transferred by conjugation at the high frequency of 1.15 × 10^−2^ per donor cell into *E. coli* J53 [107]. Therefore, it seems that in the case of *bla*_NDM_-type, conjugation plays an important role in the spread of carbapenem resistance and diverse conjugative plasmids are involved. 

Nonetheless, the *bla*_NDM-1_ can also be chromosomally located. In fact, *bla*_NDM-1_ seems to be usually flanked by two IS*Aba125* insertion sequences, a structure corresponding to the composite transposon Tn*125* [108,109,159]. This transposon was also associated with *bla*_NDM-2_, suggesting that Tn*125* is the major vehicle for the dissemination of *bla*_NDM_ genes in *A. baumannii* [110].

A very recent study showed experimental evidence of the horizontal transfer of the Tn*125* transposon carrying the *bla*_NDM-1_ gene by transduction from a carbapenem-resistant *A. baumannii* clinical strain (R2090) to a carbapenem-susceptible *A. baumannii* strain. The authors have excluded the involvement of the other three HGT mechanisms. The *bla*_NMD-1_ gene was chromosomally located and flanked by two different insertion elements (IS*Ab125* and IS*CR21*) that might explain the translocation of the gene into the strain. By full sequencing, it was possible to observe that this strain carried three intact prophages in the chromosome and it was suggested that activation of one of these prophages contributed to the transduction; nonetheless, it still remains to be tested if the phages detected are active [111]. So far, this is the first study demonstrating the possible involvement of these mobile elements in the dissemination of carbapenem resistance genes. There are about 20 *A. baumannii* phages described [161] and thus, bacteriophages may indeed contribute to the resistance evolution of *A. baumannii* under antibiotic selective pressure. 

### 6.3. Mechanisms Involved in the Movement and Dissemination of Class A β-Lactamases

#### 6.3.1. KPC-Type

KPC-type carbapenemases are rarely found in *Acinetobacter* spp. These beta-lactamases were identified for the first time in 3.4% (10/274) of clinical multidrug-resistant *A. calcoaceticus-baumannii* complex collected in 2009 in Puerto Rico. Sequencing of the *bla*_KPC_ gene revealed the variants *bla*_KPC-3_ in seven isolates, *bla*_KPC-4_, *bla*_KPC-2_ and a new variant *bla*_KPC-10_ in one isolate each [162]. A subsequent study identified 41 *A. baumannii* isolates with the *bla*_KPC_ gene (the variant was not identified) [163]. Identification of the genetic background of *bla*_KPC-3_ in *A. baumannii* demonstrated that it was embedded into Tn*4401b* located in the chromosome within a 26.5 kb fragment, which included a KQ-like element similar to one described in a *K. pneumoniae* plasmid. This data suggests the acquisition of *bla*_KPC-3_ due to an IS*Ecp1* transposition event [112]. Very recently, whole genome sequencing revealed that the *bla*_KPC-2_ gene of *A. baumannii* (Puerto Rico), assigned to the international ST2 clone, was identified on a new truncated version of transposon Tn*4401e* (tentatively named Tn*4401h*), located in the chromosome within an IncA/C plasmid fragment derived from an *Enterobacteriaceae*, probably due to the insertion sequence IS*26* [113]. This finding in the widely disseminated ST2 clone suggests further dissemination of *bla*_KPC-2_ in this species.

A study conducted in an Iranian Burn Care Center also identified the *bla*_KPC_ gene in *A. baumannii* isolates but gene transfer was not demonstrated [164].

#### 6.3.2. GES-Type

The GES enzymes are classified as extended-spectrum β-lactamases (ESBLs). However, some variants can act as carbapenemases [165]. Different levels of resistance or reduced susceptibility to carbapenems are observed with different variants [114,115,166]. Thirty-one *bla*_GES_ gene variants have been described so far [151]; only six have been reported in *A. baumannii* [2,167] (GenBank accession number NG_049127).

The first *bla*_GES_ gene reported in *A. baumannii* was the *bla*_GES-11_, which was present as a gene cassette in a class 1 integron; this mobile element was embedded into a plasmid and transfer by conjugation between two *A. baumannii* isolates was observed [114]. The *bla*_GES_ gene are embedded into class 1 integrons [116,117,118] and the majority are plasmid-located [115,116,119]. Nonetheless, chromosomal location has also been reported [116]. Transfer of the *bla*_GES_-encoding plasmid by conjugation between *A. baumannii* has been shown [115,117,119] but transfer to *E. coli* has failed [115].

The high GC content of the *bla*_GES-11_ gene as compared with the *A. baumannii* genome [114] can be seen as evidence of a different original source for this gene. The ocurrence of HGT has also been suggested due to the presence of the same *bla*_GES_ gene in *A. baumannii* isolates belonging to different genotypes [167,168].

## 7. Conclusions

The fast increase of antimicrobial resistance in the last few decades has shown the remarkable ability of the genomic plasticity of Gram-negative bacteria, leading to the ineffective control of many infectious diseases and to a global public health problem of unexpected magnitude. The emergence and dissemination of antimicrobial resistance is an ample evidence of the biological response to the enhanced exposure to toxic molecules and dynamic genetic flux among bacteria. Mutations and lateral gene transfer mechanisms are the key for bacteria genome evolution and adaptation to multiple environments. Members of the *Acinetobacter* genus are considered environmental bacteria. For unclear reasons, *A. baumannii* emerged as an important nosocomial opportunistic pathogen. Not being a normal colonizer of non-hospitalized individuals, its reservoir remains unknown. Still considered a low virulence pathogen, infecting mostly immune-debilitated hosts, it acquired enormous clinical relevance when linked to the fast acquisition of resistance to the majority of antibiotic classes, including the last therapeutic resort antimicrobials, such as carbapenems. Since the 1980s, the carbapenems were seen as a new treatment option for serious resistant infections. However, its high activity has been hindered over time by the increase of carbapenem resistance. Many isolates of *A. baumannii* are often resistant to carbapenems and, as in other Gram-negative bacteria, have at their disposal a plethora of resistance mechanisms that may act in synergy. Production of carbapenemases is undoubtedly the most common mechanism of resistance to carbapenems. Indeed, this species produce the intrinsic carbapenemase OXA-51, that despite its low efficient hydrolysis, its expression and activity can be exacerbated by the upstream IS*Aba1* or/and work in conjunction with other mechanisms, as efflux pumps, to give carbapenem resistance [169]. Nevertheless, HGT plays an important role, not yet completely understood, in the dissemination of carbapenemase determinants.

*A. baumannii* seems to have a notorious ability to survive and it can use all the mechanisms of HGT and mobile genetic elements to spread resistance genes (Table 1). Understanding the extent of carbapenem resistance and how its mobilization takes place is essential to control the dissemination of these genes. 

As in other Gram-negative bacteria, conjugation appears to have the greatest influence in the spread of carbapenem resistance determinants. Some of the CHDLs are disseminated by conjugative plasmids and the MBL *bla*_NDM_ gene seems to be often located in different plasmids of diverse sizes. However, most reports associated *bla*_NDM_-type with Tn*125*, suggesting the intracellular ”jumping” of the genetic elementintochromosome and to other plasmids of the new host bacteria. Indeed, *bla*_NDM_-type inserted in Tn*125* has been found integrated in the chromosome [109], supporting the idea of the high dynamic of this gene, which might explain its global widespread in diverse genetic platforms.

For the first time, transduction has been recently suggested to occur as a mechanism of the spread of *bla*_NDM_ genes in *A. baumannii* [111]. Not very widely reported, it is obvious that transduction is an important mechanism of lateral gene transfer when analyzing the genomes of diverse bacteria [170]. Moreover, many bacteria strains gained their virulence (production of some toxins) by the integration of stable prophage in their chromosomal DNA [170,171]. Therefore, it is evident that phage-mediated transduction can contribute to the transfer of resistance genes in *A. baumannii*. Nevertheless, phages are generally specific to the bacteria species [172], which might be a limitation for the spread to other species; however, interspecies transfer of genetic material by transduction was observed [173].

Natural transformation is usually an underestimated mechanism of lateral gene transfer. A barrier is that bacteria need to be naturally competent (and probably why it is thought that it occurs at a low frequency), i.e., to have protein-encoded genes necessary for the uptake of naked DNA, and then, stable integration in the chromosome (or reconstitution of plasmids in the bacterial cytoplasm) [174]. Some members of *Acinetobacter* spp. are naturally competent. It was demonstrated that integrons carrying the MBL *bla*_IMP-5_ gene can be inserted in the chromosome of *A. baylyi*, a naturally competent species, with a negligible biological cost to the recipient cell [97]. Some MBLs, especially from the IMP- and VIM-type families, are part of integrons and many of these isolates do not carry plasmids, suggesting that conjugation is not the involved mechanism (unless they lost the plasmid after transposition to the chromosome). IMP and VIM were the first metallo carbapenemases to be described and despite the increasing number of variants since then, they are not widely spread in *A. baumannii*. Natural transformation may contribute to their dissemination, even if at an initially low rate, which might be followed by high frequency dissemination [97]. This could explain the movement of integrons in the environment among diverse species [175,176] but not in clinical settings. However, recently clinical *A. baumannii* [57,58] and *A. nosocomialis* [177,178] strains were reported as naturally competent in very specific conditions, raising the hypothesis of a role of natural transformation in the resistance dissemination among clinical bacteria. Interestingly, this feature does not appear to be present in all members of the species or be characteristic of a clonal lineage of *A. baumannii*. Moreover, experimental conditions have a critical impact on the acquisition of foreign DNA [58,179]. Nevertheless, this is a mechanism that deserves to be explored with clinical strains and specifically what environmental conditions might influence the acquisition of naked exogenous DNA.

Another unusual reported HGT mechanism is related with the production of OMVs. The mechanisms of vesicle-mediated DNA delivery are not completely understood. A possibility is that competence proteins play a role in the uptake of DNA delivered by OMVs, since vesiculants (transformed cells obtained by vesicle-mediated gene transfer) were not formed with *com*- deficient mutants in *A. baylyi*. Interestingly, sub-inhibitory concentrations of gentamicin increased 10 times the transfer of plasmid DNA by OMVs, which might be explained by the altered surface potential by gentamicin-treated population [180]. 

Recent studies confirmed that *A. baumannii* are able to produce OMVs. It was demonstrated that OMVs released by *A. baumannii* are able to transfer *bla*_OXA-24_ [59], which reinforces the notion that the mediated-OMV mechanism must be taken into account for the dissemination of carbapenemase genes in this pathogenic species. Another study reported the extracellular delivery of OXA-58 in OMVs that shelter cells (even of other species) to the noxious action of carbapenems [145,146]. Not being a way of gene transfer, it is a way of protecting a diverse population of the toxic effects of antibiotics. The mechanisms of delivery from OMVs are still not very clear but the hypothesis suggested by Fulsundar et al. [180] and the recent findings of clinical competent strains, opens new fields of research.

Overall, the dissemination of carbapenemases in *A. baumannii* illustrates the complexity of the micro-dynamic flow of genes and their associated mobile genetic elements. Understanding these genetic mechanisms will allow gains and insights into the epidemiology of resistance genes and to predicting its spread, in order to act in the prevention of dissemination of antimicrobial resistance genes.

## Figures and Tables

**Table 1:** Carbapenemase Genes in *Acinetobacter baumannii*: Genetic Location, Horizontal Gene Transfer Mechanisms, Genetic Context and Movement of Mobile Genetic.

Carbapenemases	*bla* Gene Type	Resistance Origin	Location	Intercelullar Transfer (HGT) ^1^	Genetic Context	Intracellular Movement	References
CHDLs ^2^	OXA-51	Intrinsic	Mainly chromosomal;		Sometimes, IS*Aba1-bla*_oxa-51_-like		[33,35]
			plasmid		Tn*6080*	Transposition	[34]
	OXA-23	Acquired	Chromosome and plasmid	Conjugation	Genomic islands; Transposons; Insertion sequences	Transposition	[62,63,64,65,66,67,68,69,70,71,72,73]
	OXA-40/24	Acquired	Chromosome and plasmid	Conjugation? ^5^	Self-transmissible plasmid belonging to replicon group GR6		[45,74,75,76,77]
					and in plasmids containing mob genes		[52,78]
					Flanked by conserved inverted repeats homologous to XerC/XerD, recombinases’ targets	Site-specific recombination	[77,78,79,80]
				OMVs ^4^-mediated transfer			[59]
	OXA-58	Acquired	Mostly plasmidic	Conjugation? ^5^	Self-conjugative plasmid belonging to replicon group GR6		[52,67,81,82,83]
					Insertion sequences		[3,50,84,85]
					Flanked by two repeated sequences, Re27	Homologous recombination	[84]
					Flanked by two IS*26* leading to duplication of IS*Aba2*/IS*Aba3*-*bla*_OXA-58_-IS*Aba3*	Transposition	[86]
	OXA-143	Acquired	Plasmid		Flanked by two *rep* genes	Homologous recombination	[45]
					XerC/XerD recognition site	Site-specific recombination	[87]
	OXA-235	Acquired	Chromosome and plasmid		Flanked by two IS*Aba1*	Transposition	[46]
MBLs ^3^	IMP	Acquired	Chromosome and plasmid	Conjugation	Many inserted in class 1 integrons		[88,89,90,91,92,93]
					Gene not embedded into integrons		[94]
					Class 1 integron flanked by MITEs ^6^	Transposition	[95,96]
				Natural transformation	Chromosomal class 1 integron	Homologous recombination	[89,97]
	VIM	Acquired	Chromosome		Class 1 and 2 integrons		[98,99,100,101,102], GenBank Ac. nr. LC107606
	NDM	Acquired	Mostly plasmidic;	Conjugation	Associated to composite transposon Tn*125*	Transposition	[103,104,105,106,107,108,109,110]
			chromosome	Transduction	Flanked by IS*Aba125* and IS*CR21*		[111]
Class A β-lactamases	KPC	Acquired	Chromosome		Tn*4401b* embedded into KQ-like element	Transposition	[112]
					(Novel truncated version of) Tn*4401e*		[113]
	GES	Acquired	Chromosome and plasmid	Conjugation	Class 1 integrons		[114,115,116,117,118,119]

^1^ HGT, Horizontal gene transfer; ^2^ CHDLs, Carbapenem-hydrolysing oxacillinases; ^3^ MBLs, Metallo-β-lactamases; ^4^ OMVs, Outer-membrane vesicles; ^5^ Failed demonstration [50,51,76,78,120]; ^6^ MITEs, Miniature inverted repeat transposable elements.
